# A Case of Synocha

**Published:** 1801-03

**Authors:** James Moore

**Affiliations:** Grosvenor Street


### *A Cafe of Synocha, communicated by* Mr. James Moore, *Surgeon.*

SYNOCHA, or pure inflammatory fever, is a difeafe fo rare in this country, that many experienced practitioners have doubted its exiftence. I think the following Cafe, which I lately attended, is unqueftionably an example of it.

J. H. is thirty-one years of age, he is a tall ftout man, of a florid complexion, and of a full fanguine habit. From a particular caufe, he has for above a year laboured under a depreffion of fpirits, and unfortunately he was lately terrified to a great degree. As his mind continued in a ftate of alarm, there is reafon to believe that this was the remote caufe of the fever which enfued.

The induftrious Hoffman, in enumerating the caufes of fevers, mentions, firft, "vehementes animi commotiones, terror imprimis et ira."

This young man, though haraffed by thefe terrible paffions, endeavoured to fupprefs all appearance of them; and as he was in the country, and did not complain when he firft felt himfelf indifpofed, I cannot with certainty fix the firft day of the fever. Indeed, this in many cafes is impoffible, the beginning of difeafes being often imperceptible.

However, according to the beft conjecture I can make, the fever commenced October 29th, when he perceived a chillinefs all over his body: But for feveral days before he was unwell, and had fallen off in his appetite.

The fecond day of the fever, ficknefs occurred, though not in fuch a degree as to excite vomiting, and in the night he broke out into a profufe perfpiration.

The third day, the perfpiration continuing, he kept his bed, and complained of head-ach. An opening medicine was given him.

The

The fourth morning he was better, and ſat up in the day; but grew worſe towards the evening. He ſtarted from bed during the night, and was kept in a continual ſtate of terror, from believing he ſaw frightful apparitions.

The fifth day he dreſſed himſelf, got upon horſe-back, and rode to town, which was a diſtance of twelve miles. He complained very little, but was thought to be in a ſtrange ſtate.

The ſixth day I was conſulted. I found him up, and when I inquired how he was, he told me he had only a pain in his forehead. His face was redder than uſual, and his eyes were ſlightly inflamed. The expreſſion of his countenance denoted ſurpriſe; and the anſwers to the queſtions I put to him, marked a confuſion of intellect.

His pulſe was ſtrong, hard, and beat 88 ſtrokes in a minute. The ſkin was hot, the tongue was moiſt and whitiſh, the urine red, with a dark ſediment; the bowels regular.

He was put to bed, and as the delirium augmented, it was found neceſſary to guard him carefully.

The diſeaſe increaſed, though with occaſional remiſſions, for four days: His pulſe was always ſtrong and regular, and once was perceived as high as 96; his ſkin felt hot, and rather moiſt; he was diſpoſed to conſtipation, was thirſty, and ſhewed no nauſea or want of appetite, but ſwallowed readily whatever was given him.

On the tenth day he was quite furious, and could hardly be kept in bed, though ſtrapped down, and reſtrained by two ſtrong men. That night a profuſe ſweat broke out, and he became tranquil.

The eleventh day I found him perſpiring freely. His pulſe was ſoftened, and diminiſhed in frequency, and his anſwers were rational.

This proved the criſis of the fever; for, on the twelfth morning, his pulſe had ſunk to 80, and his only complaint was weakneſs.

The treatment employed during the five days he was under my charge, conſiſted ſimply of two purgatives, and a draught containing one-fourth of a grain of tartar emetic, and two drams of the aqua ammoniæ acetatæ, which was exhibited regularly every ſix hours.

This, I imagine, contributed to excite the critical perſpiration.

I did not venture on bleeding, becauſe it was the ſixth day of the fever before I ſaw him.

His diet conſiſted of liquids, ſlightly nutritious.

The definition of Synocha given by Dr. Cullen, is "Calor plurimum auctus, pulsus frequens, validus et durus; urina rubra sensorii functiones parum turbatæ."

This case differed in the last characteristic; but as Dr. Cullen acknowledged that he never saw the disease, he may have erred in the description.

It is also probable, that the mental derangement in this instance was much greater than usual.

This case was so strongly marked, that there could be little danger, without gross inattention, of mistaking it for a fever of the typhoid kind.

The loss of strength was so slight, that the patient rode twelve miles on the fifth day, without appearing fatigued, or going to bed afterwards: And when the disease left him altogether, the debility was much less than what occurs after fevers in general.

The natural functions were little disturbed: His thirst was not excessive; and he took whatever was allowed him without disgust.

The pulse was strong and hard, the skin hot and soft; every one of which particulars is the reverse of what occurs in Typhus.

And the tongue, instead of having a dry, red, brown, or black appearance, was always moist, and rather white.

As most of the functions of the body were so little disordered, delirium was unexpected.

It commenced so early as the fourth night, and continued till the crisis with augmenting violence. Perhaps the moral causes, which it is believed, operated in exciting the disease, contributed to this effect.

The indications in this fever are very opposite from those of Typhus, it is therefore of the utmost importance that they should be discriminated.

Synocha certainly very much resembles the symptomatic fever attendant upon phlegmon; and therefore, it has not unnaturally been termed the inflammatory fever.

The common ephemera is undoubtedly of the same species, which, notwithstanding its name, often continues three days:

And the Synocha seems to me precisely the same malady, in a more violent degree, and running on for a longer period.

As many cases similar to the above have been narrated by authors, it appears strange that the reality of this disease should be now questioned.

But the attempt to simplify diseases, and particularly fevers, has, I think, been carried to an erroneous length.

The

The ſpecies that are common in any country, are perhaps not numerous; but it is clear, from the various accounts we receive, that fevers have different ſymptoms, and require a different treatment in every part of the globe.

This iſland ſeems moſt fortunate in this particular; for in no country, however our climate is decried, are fevers ſo mild and ſo little dangerous. Among the poor, who are lodged in confined apartments, and who are too frequently neglected, it is true that fatal caſes are common.

But among thoſe who are comfortably lodged, and who have the means of obtaining early and proper aſſiſtance, except where the conſtitution is naturally bad, or is worn out with old age, fevers are rarely mortal.

*Groſvenor Street,*
*Jan.* 3, 1801.

JAMES MOORE.

